# Characterization of Antigen-Specific B Cells Using Nominal Antigen-Coated Flow-Beads

**DOI:** 10.1371/journal.pone.0084273

**Published:** 2013-12-30

**Authors:** Nicolas Degauque, Annie Elong Ngono, Ahmed Akl, Maud Lepetit, Romain Crochette, Magali Giral, Julie Lepourry, Annaick Pallier, Stéphanie Castagnet, Emilie Dugast, Cécile Guillot-Gueguen, Marylène Jacq-Foucher, Xavier Saulquin, Anne Cesbron, David Laplaud, Arnaud Nicot, Sophie Brouard, Jean-Paul Soulillou

**Affiliations:** 1 INSERM, UMR 1064, Nantes, France; 2 CHU de Nantes, ITUN, Nantes, France; 3 Université de Nantes, Faculté de Médecine, Nantes, France; 4 INSERM, CIC 004, Nantes, France; 5 INSERM, UMR892, Nantes, France; 6 Etablissement Français du Sang, Laboratoire HLA, Nantes, France; Institut National de la Santé et de la Recherche Médicale U 872, France

## Abstract

In order to characterize the reactivity of B cells against nominal antigens, a method based on the coupling of antigens onto the surface of fluorescent core polystyrene beads was developed. We first demonstrate that murine B cells with a human MOG-specific BCR are able to interact with MOG-coated beads and do not recognize beads coated with human albumin or pp65. B cells purified from human healthy volunteer blood or immunized individuals were tested for their ability to interact with various nominal antigens, including viral, vaccine, self and alloantigens, chosen for their usefulness in studying a variety of pathological processes. A substantial amount of B cells binding self-antigen MOG-coated beads can be detected in normal blood. Furthermore, greater frequencies of B cell against anti-Tetanic Toxin or anti-EBNA1 were observed in primed individuals. This method can reveal increased frequencies of anti-HLA committed B cells in patients with circulating anti-HLA antibodies compared to unsensitized patients and normal individuals. Of interest, those specific CD19 cells were preferentially identified within CD27^−^IgD^+^ (i-e naïve) subset. These observations suggest that a broad range of medical situations could benefit from a tool that allows the detection, the quantification and the characterization of antigen-specific blood B cells.

## Introduction

The crucial role of B cells in a number of autoimmune diseases, such as multiple sclerosis [1] and rheumatoid arthritis [2], has been recently highlighted through the study of anti-CD20 in clinic. Having access to specific antigen committed blood B cells in humans would be an important step towards better understanding B cells’ potential role in autoimmunity and responses against infectious agents and allotransplants. B cells are not only plasmocyte progenitors, but also display regulatory functions [3], [4], are good presenting cells [5] and can have direct cytotoxic effects[6]–[8]. Mechanisms shaping the early B cell repertoire rely predominantly on receptor editing and anergy, and not on deletion [9], [10]. However, in humans a substantial frequency of mature circulating B cells still show some degree of autoreactivity and or polyreactivity, which survives the first checkpoint of B cell repertoire maturation [11], and persisting autoreactive B cells in the mature repertoire [12]. There is thus a continuous need for effective regulation – mostly from T_REG_– to avoid any deleterious reaction.

In human, the analysis of autoreactive B cell frequency has been most often indirectly approached using the reactivity of antibodies produced *in vitro* in B cell culture supernatants in limiting dilution conditions [13], where it seems that tools identifying committed B cells by direct interaction would be more effective. A number of such direct interaction approaches have been developed such as the use of modified tetramers that consist of a R-PE-labeled streptavidin core and four biotinylated proteins [14]. The main limitation of such an approach is the heterogeneous binding of B cells. B cells will not only bind to the target protein but also to the fluorescent molecule (i-e PE) and biotin epitopes within the tetramer. To circumvent this problem, a concomitant use of another tetramer (conjugated to a different fluorochrome) is needed to exclude unspecific binding. In addition, such a method may face technical difficulties in achieving a stereotyped labeling of the reagents, which may vary from batch to batch.

In this report, we used fluorescent Bio-plex COOH beads that contain a fluorescent internal core and can be covalently linked to any protein. A broad variety of antigens can be analyzed simultaneously through varying the ratio of two fluorescent molecules within the bead internal core. The strategy was first assessed using B cells purified from 8.18-C5 transgenic mice expressing human anti-MOG BCR [15]. B cells purified from healthy human blood and immunized individuals were then tested for their ability to interact with various nominal antigens, including viral, vaccine, self and alloantigens, all of which may have some usefulness to the study of various pathological processes. For instance, we show increased frequencies of anti HLA committed B cells in patients with circulating anti HLA antibodies compared to unsensitized patients or normal individuals. We also show that, similarly to T cells [16], [17], a substantial amount of B cell binding self-antigen MOG coated beads can be detected in normal individual blood, confirming the permissivity of the first B cell tolerogenic checkpoint in humans. Furthermore we show that there is a high frequency of blood B cells against anti-Tetanic Toxin or anti-EBNA1 in primed individuals. Finally, B cells could be depleted from MOG specific B cells and this later fraction could be enriched by more than 40 fold. These observations suggest that a broad range of medical situations could be benefit from a tool that allows the detection, the quantification and the characterization of blood antigen-specific B cells.

## Materials and Methods

### Subjects and Ethics Statement

The University Hospital Ethical Committee and the Committee for the Protection of Patients from Biological Risks approved the study. All kidney transplant patients (n = 31) and healthy volunteers (n = 38) included for study gave written informed consent (**Table 1** **and** **2**). Two sets of kidney transplant recipients were recruited based on the use of single HLA-A*0201 coated beads (**Table 1**) and single HLA class I antigen coated beads (**Table 2**). **(** **Table 1** **).** Kidney transplant recipients with specific anti-HLA-A*0201 antibodies (Luminex) and, biopsy-proven or not, antibody mediated rejection [18] (ABMR; n = 10). Eight non-sensitized kidney transplant recipients with stable graft function under standard immunosuppression (serum creatinine<150 µmol/L and proteinuria <1 g/24 h and with less than 20% change in the values between the two previous 6-month follow-up visits). **(** **Table 2** **).** Thirteen transplant patients under standard immunosuppression with kidney graft dysfunction (serum creatinine >150 µmol/L) during the study follow-up periods, who had developed anti-HLA class I antibodies.

**Table 1:** Summary of demographic and clinical characteristics of patients analyzed using single HLA-A*0201 antigen coated beads.

Demographic data	Non-sensitized recipients (n = 8)	Sensitized recipients (n = 10)
Recipient Age (years)	47 (30–70)	42 (19–64)
Recipient Gender (M/F)	5/4 (55%)	3/7 (30%)
Donor Age (years)	38 (16–73)	47 (19–63)
Donor Gender (M/F)	7/1 (87%)	5/4 (55%)
**Clinical data**		
Time post-transplantation (years)	4.55 (0.64–14.38)	6.52 (0.73–17.59)
HLA mismatch (HLA-A, -B, -DR) >4	6	3
HLA Class I Ab (MFI >1500)	0/8	10/10
Class I DSA (MFI >1500)	0/8	1/10
Creatinemia (umol/L)	125 (87–156)	110 (62–182)
**Stage of chronic kidney disease**		
Stage 1–2	8	10
Stage 3–5		
**Induction Therapy**		
Monoclonal Ab/Polyclonal Ab/None	5/2/1	0/9/1
**Maintenance Therapy**		
FK/CSA/mTOR Inhibitors/None	6/2/0/0	6/4/0/0
Corticotherapy	6	9

**Table 2:** Summary of demographic and clinical characteristics of patients analyzed using single HLA class I antigen coated beads.

**Demographic data**	
Recipient Age (years)	52 (21–73)
Recipient Gender (M/F)	5/8 (38%)
Donor Age (years)	58 (27–72)
Donor Gender (M/F)	5/6 (45%)
**Clinical data**	
Time post-transplantation (years)	1.99 (0.00–19.78)
HLA mismatch (HLA-A, -B, -DR) >4	10
HLA Class I Ab (MFI >1500)	13/13
Class I DSA (MFI >1500)	5/13
Creatinemia (umol/L)	240 (62;947)
**Stage of chronic kidney disease**	
Stage 1–2	2
Stage 3–5	10
**Induction Therapy**	
Monoclonal Ab/Polyclonal Ab/None	7/5/0
**Maintenance Therapy**	
FK/CSA/mTOR Inhibitors/None	9/1/3/0
Corticotherapy	10

Values are median (min-max) for continuous variables or number of patients (%) for categorical variables. *one missing data and ¶ more than one missing data. Anti-class I Ab analysis, using Luminex HD, was performed for all but 1 patient.

### Blood Samples and B Cell Isolation

Peripheral blood mononuclear cells (PBMC) were separated on a Ficoll gradient layer and either frozen in DMSO-10% autologous serum or used to purify CD19^+^ B cells using the human B Cell Isolation Kit (Miltenyi Biotech) according to the manufacturer’s instructions.

### Coupling of Proteins to Fluorescent Beads

20 µg of recombinant human MOG_1–125_ (extracellular domain of human MOG along with a 6x His tag expressed in E.coli.; Eurogentec), human albumin (LFB), CMV pp65 peptide (SVLGPISGHVLKAVF; Eurogentec), EBNA-1 (1–90 and 408–498 amino acids; Prospec) and non-toxic TT C-fragment from clostridium tetani (Sigma) proteins were coupled to fluorescent Bio-plex COOH beads (Bio-Rad) or magnetic fluorescent Bio-plex COOH beads (Bio-Rad) according to the Bio-Plex Amine Coupling Kit (Bio-Rad) instructions. The efficiency of the coupling reaction was assessed systematically by flow cytometry.

### Magnetic Enrichment of Ag-specific B Cells

3×10^6^ purified CD19^+^ cells from MS patients were incubated with 5 uL of MOG_1–125_ coated magnetic fluorescent Bio-plex COOH beads for 60′ at 4°C in a final volume of 100 uL of PBS/2%FCS/2 mM EDTA buffer. After incubation, antigen-specific B cells were subjected to two-round of positive selection by placing the eppendorf in a Dynal® MPC-S magnet for 1′, removing the supernatant and addition of 1 mL of PBS/2%FCS/2 mM EDTA buffer.

### Quantification of Ag-specific B Cells in Mice

Dissociated splenocytes from transgeneic mice [19] (generous gift from Dr. G. Krishnamoorthy and H. Wekerle, Max Planck Institute of Neurobiology) were frozen in DMSO-10% autologous serum. The transgenic mice, generated by Litzenburger *et al*., express the VDJ region of the MOG-specific H chain from the hybridoma 8.18-C5 [15]. To quantify antigen specific B cells, splenocytes were stained with CD19-PE (1D3), incubated with beads (60′ at 4°C in the dark) and events were acquired on a flow cytometer LSR II (BD Biosciences). DAPI was systematically used as viability marker.

To evaluate the specificity of transgenic B cells, spleen cells stained with anti-CD19 mAb were pre-incubated for 30′ with 20 µg of free proteins (MOG_1–125_, albumin or pp65), washed before adding beads coupled with MOG_1–125_ as previously described. In order to establish the affinity of beads-B cells interactions, increasing concentrations of MOG_1–125_ were used to prevent the binding between B cells and MOG_1–125_ -coated beads.

### Identification of Nominal Antigen-specific B Cells

Frozen PBMC or purified B cells were used to analyze the B cell reactivity toward single antigen HLA class I coated beads (One Lambda) or custom nominal antigen coated beads. The reactivity toward 97 HLA class I antigens (31 HLA-A, 50 HLA-B and 16 HLA-Cw alleles) was tested simultaneously using beads with different ratio of 2 fluorochromes. Similar results were obtained using PBMC or purified B cells. However, to reduce the time of acquisition and the amount of antigen-coated beads, purified B cells were preferentially used. 5×10^6^ PBMCs or at least 5×10^4^ purified B cells were surface stained with antibodies specific for CD19 (HIB19) and IgD (IA6-2) and CD38 (HIT2); or CD24 (ML5) and CD38 (HIT2); or CD27 (M-T271) and IgD (IA6-2). DAPI was used to exclude dead cells from the analysis. The cells were analyzed with a LSRII flow cytometer. After surface staining, the cells were resuspended in 100 uL of PBS 0.5% bovine serum albumin (BSA), and 2 mM EDTA. Nominal antigen coated beads were added and incubated at room temperature for 20′ in the dark. 300 uL of PBS/BSA/EDTA was added before the acquisition. All the antibodies were from BD Biosciences. Frequencies of antigen specific B cells below 0.01% were discarded.

### Inhibition Assay Using HLA-A*0201 Multimer

CD19 stained purified B cells were incubated with a cocktail of HLA-A*0201 multimer (HLA-A*0201/MP_58–66_, HLA-A*0201/HCw1, HLA-A*0201/pp65; synthesized as previously described [20]) and then with 2.5 uL of single HLA-class I antigens (at room temperature for 20 min in the dark). 300 uL of PBS/BSA/EDTA was added before the acquisition.

### Depletion of B Cell Specific for Single-HLA Class I Antigen-coated Beads

CD19^+^DAPI^−^ cells that did not recognized any of the HLA class I molecules were isolated using an ARIA FACS-sorter from 2 healthy volunteers and cultured for 4 days in complete RPMI medium supplemented with the following activation cocktail (anti-F(ab)’2 2 µg/mL; CD40L 50 ng/mL; enhancer 5 µg/mL; ODN2006 2.5 µg/mL; IL-2 50 U/mL). After 4 days of culture, the cells were recovered, incubated with HLA-class I coated beads for 20 min at room temperature before cell acquisition.

### Statistical Methods

Comparison was performed using a Mann-Whitney test or a Kruskall-Wallis test followed by a Dunn’s post-hoc test, using Graphpad Prism 5. P-values below 0.05 were considered statistically significant.

## Results

### Identification of CD19 Cells Reactive to Antigen-coated Beads

**Figure 1:**
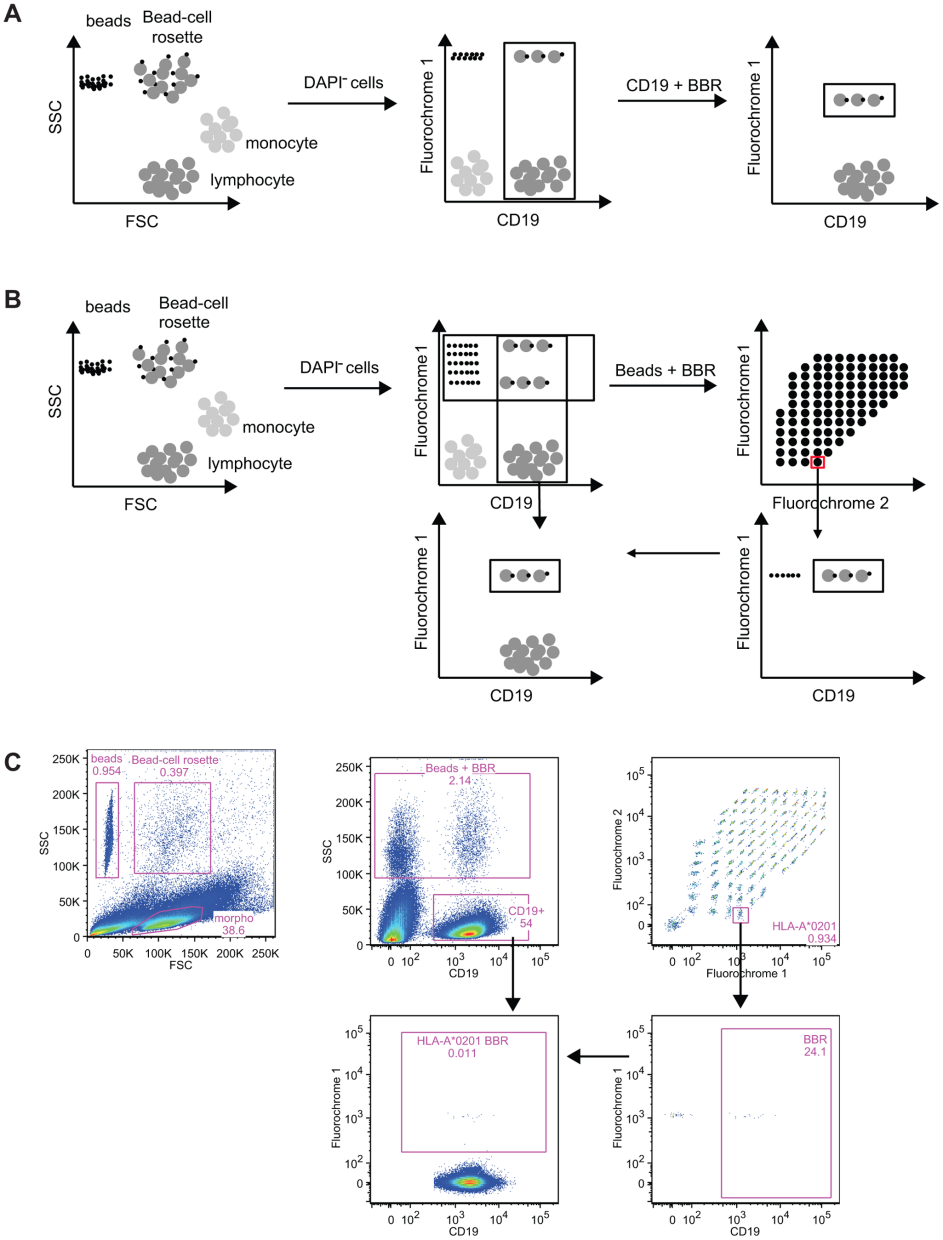
Principle of the method of identification of antigen-specific B cells. After co-incubation, lymphocytes, antigen covered beads and the beads’ B cell rosettes are gated based on their forward scatter and side scatter. After exclusion of the DAPI+ cells, B cells and beads-B cell rosettes are identified based on CD19 expression and the beads’ internal fluorochrome. Specificity of B cell recognition is determined by gating on beads and beads’ B cell rosettes (**A**) or after the identification of the nominal antigen through the use of the unique ratio of the two internal fluorochromes (**B**). In the latter, for each nominal antigen, a gate that encompassed beads and B cell rosettes is created followed by the identification of the B cells. Frequency of B cells bound to HLA class I of interest is finally evaluated. Bead-based method allows the detection of antigen-specific B cells. (**C**). An example of the identification of beads, Bead-cell rosette and lymphocyte is shown. After exclusion of dead cells, the use of the marker CD19 allows the identification of B lymphocyte and a mix of beads and BBR. Thanks to the ratio of two fluorochromes, antigen coated on the beads can be then identified. Beads are excluded using the expression of CD19. A Boolean gate is used to assess the frequency of B cells specific of a given antigen within the whole B cell population.

In order to characterize the reactivity of B cells against nominal antigens, a method based on the coupling of antigens onto the surface of fluorescent core polystyrene beads was developed. Such beads are routinely used to identify anti-HLA antibodies in the serum of kidney transplant recipients [21], [22] and, more recently, to some anti-self-antigen antibodies (MPO, ANCA, PR3) [23]. The principle of this method applied to the detection of B cells committed to a given antigen is shown in Figure 1 as well as an example (Figure 1C). The reactivity of B cells against a single (Figure 1A) or multiple antigens (beads of different fluorescence coated with a nominal antigen; Figure 1B) can be assessed. After co-incubation of the cells with single antigen-coated beads, cells that bound to the beads are first identified based on their forward and side scatter. Bead-B cell rosettes (BBR) exhibit both the features of antigen-coated beads (very low FSC and high SSC) and of B cells (intermediate FSC and low SSC). After the exclusion of DAPI+ cells, B cells that recognized single antigen-coated beads are identified in the upper right gate. The frequency of B cells interacting with single antigen coated beads can then be obtained after the exclusion of beads that do not interact with B cells (Figure 1A). When multiple reactivity is analyzed at the same time, as in for HLA alleles (Figure 1B), the frequency of B cells specific to beads coated with a given antigen is evaluated after the successive gating of: 1) single antigen-coated beads – CD19^+^ cells rosettes, 2) the target antigen specificity (using the unique grade of 2 fluorochromes), and, 3) B cells bound to the specific antigen-coated beads. Knowing the number of B cells within the sample, the frequency of B cells specific to a given HLA Class I antigen-coated bead can be estimated. In the following sections, we show that this test allows the enumeration of the frequency of B cells committed against a variety of antigens, ranging from autologous, alloantigens, viral to vaccination determinants.

### Bead-based Method Allows the Measurement of Antigen-specific B Cells Frequencies

**Figure 2:**
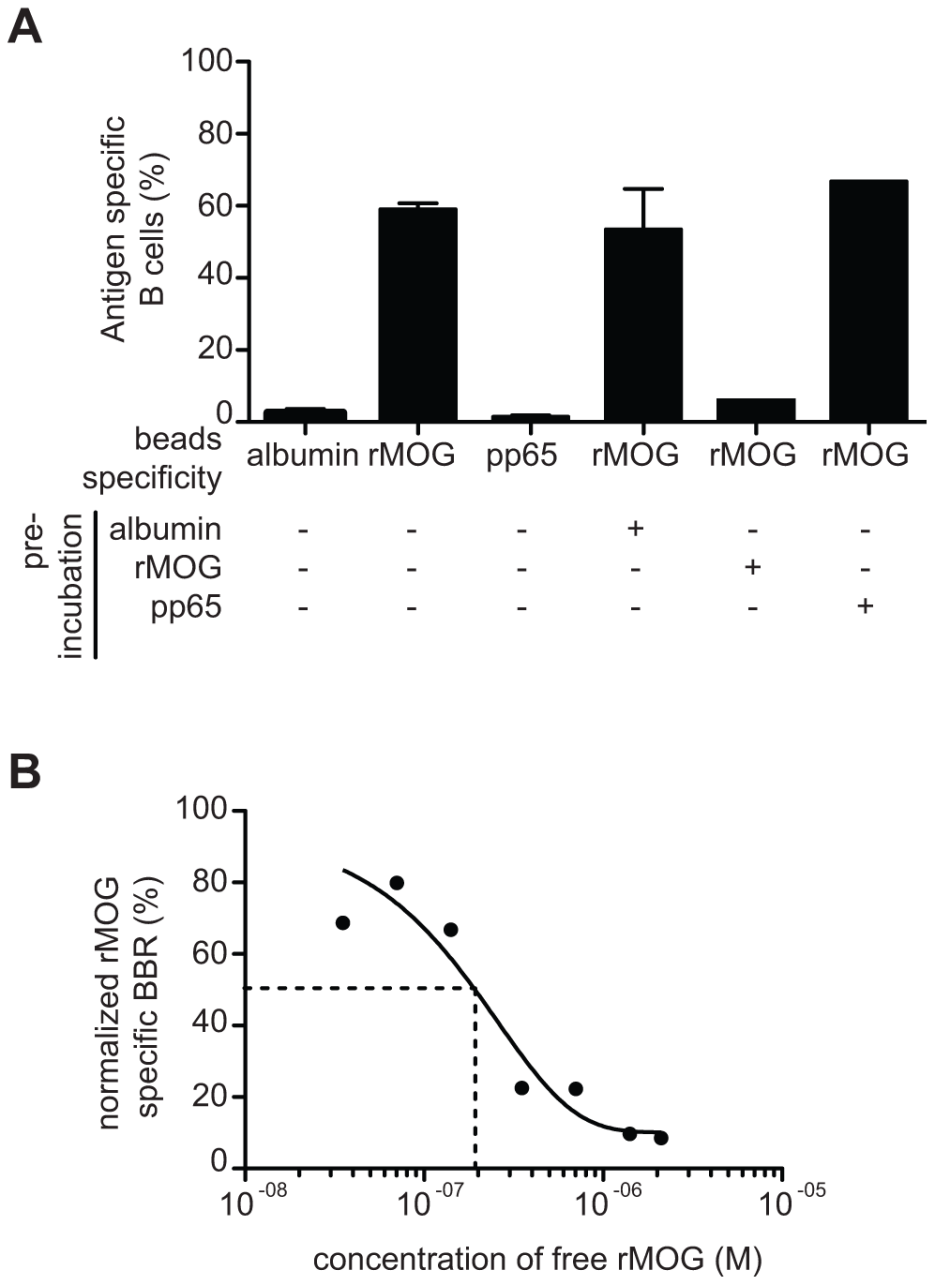
Bead-based method allows the detection of antigen-specific B cells. (**A**) B cells purified from Tg mice were incubated with human albumin, MOG_1–125_ or pp65 coated beads and the frequency of antigen specific B cells was quantified. The B cells were preincubated with soluble human Albumin, MOG_1–125_ or pp65 before incubation with MOG_1–125_ coated beads. Data are presented as mean ± sem **B**). B cells purified from Tg mice were preincubated with increasing doses of soluble MOG_1–125_ before incubation with MOG_1–125_ coated beads. The experiments were repeated 3 times and similar results were obtained.

Splenocytes from 8.18-C5 mice [15] expressing a transgenic human anti-MOG BCR were used to validate the principle of the method. When transgenic B cells were co-incubated with MOG-coated beads, 59.07±2.85% of B cells bound to the beads (Figure 2A), whereas binding to human albumin- and pp65-coated beads was low (2.58±1.83% and 1.39±0.78 respectively). The addition of the soluble MOG_1–125_ before the incubation with MOG_1–125_ -coated beads prevented the interaction with MOG coated beads whereas the frequency of MOG_1–125_ specific B cells was unchanged in the presence of soluble human albumin or pp65 (Figure 2A). To better assess the degree of affinity of the BCR toward MOG, B cells were then preincubated with increasing concentrations of soluble MOG_1–125_ (3.5×10^−8^ to 2.1×10^−6^ M) before MOG_1–125_ -coated bead labeling. Figure 2B shows a dose dependent decrease of MOG specific B cells when increasing amounts of soluble MOG_1–125_ is added. Incubation with 1.8×10^−7^ M of free rMOG_1–125_ was needed to reduce the frequency of rMOG_1–125_ specific BBR by 50%, suggesting that the affinity of rMOG1–125 and BCR interaction is rather low. Taken together, the results show that an antigen specific interaction between B cells and beads coated with a nominal antigen can be detected and quantified.

### CD19^+^ B Cells from Healthy Individuals Exhibit a Broad Frequency Range for Reaction Against Self-antigens, Vaccine or Viral Proteins

**Figure 3:**
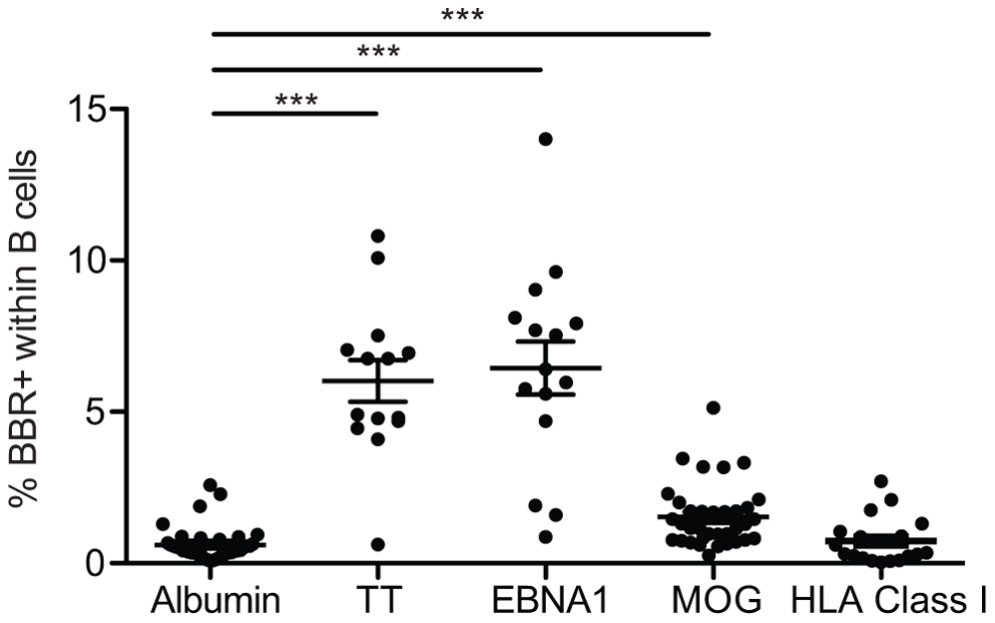
B cells from healthy volunteers exhibit a broad range of reactivity. Purified B cells from healthy volunteers were tested for their reactivity to albumin (n = 38), Tetanus Toxin (n = 14), EBNA1 (n = 15), MOG_1–125_ (n = 38) and a panel of 97 HLA class I molecules (n = 19). ***p<0.001 (Kruskall-Wallis follow by a Dunn’s post hoc test using albumin settings as reference group).

The reactivity of CD19^+^ cells purified from normal individuals was then assessed against different antigens, including beads coated with a self-antigen (MOG_1–125_), a virus antigen (EBNA1), an antigen used for vaccination (Tetanus Toxin, TT) and HLA Class I molecules. Albumin was used as a control antigen. A gradient of BBR frequency was observed ranging from low frequency when HLA Class I molecules (in unprimed individuals) and albumin were used to a high frequency when TT or EBNA1 (or HLA primed individuals – see next sections) were used. Indeed, the frequency of B cells from unprimed male subjects interacting with the panel of HLA class I molecules was similar to the frequency of B cells interacting with Albumin (mean±sem; 0.73±0.17 vs. 0.61±0.09 respectively, ns; Figure 3). Of note, the frequency of B cells interacting with non-coated beads was as low as 0.014±0.005. As discussed below, the BBR frequency for proteins against which normal individuals were not immunized is consistent with the frequency of circulating B cells producing self/poly reactive antibodies and thus escaping the central checkpoint during maturation [11], [24]. The frequency against self-antigen MOG_1–125_ was 2.5 times higher than Albumin (mean±sem; 1.53±0.16 vs. 0.61±0.09 respectively; p<0.001; Figure 3), comparable to the substantial frequency of T cells committed against MOG_1–125_ in circulating T cells of normal individuals [16], [17], [25]. However, reactivity against TT and EBNA1 coated beads was 9.8 and 10.5 times higher than the control antigen (mean±sem; 6.02±0.69 vs. 6.44±0.88 vs. 0.61±0.09%, respectively. p<0.001; Figure 3). Finally, the ability of B and T cells purified from 3 male individuals to interact with Albumin-coated beads was similar (mean±sem; 0.10±0.03 vs. 0.16±0.03%, respectively; data not shown): B cells, but not T cells, were able to interact with self-antigen MOG_1–125_ (mean±sem; 0.63±0.15 vs. 0.04±0.01%, respectively).

### CD19^+^ B Cells Reacting Against Nominal Antigens and Unbound CD19^+^ Fraction can be Efficiently Separated

**Figure 4:**
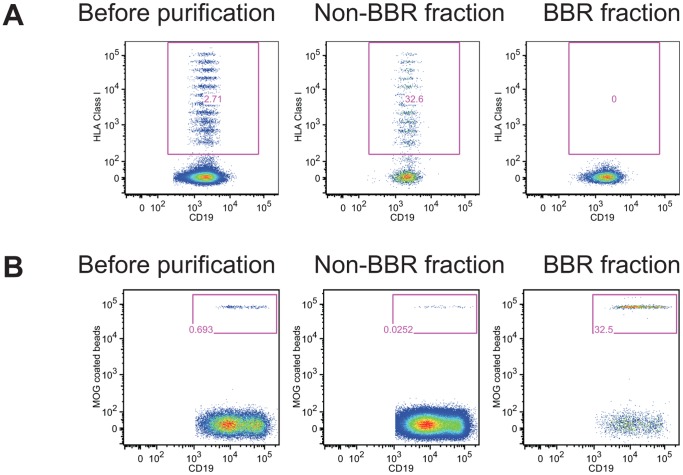
Separation of B cells interacting with nominal antigen and unbound B cells. Purified B cells were incubated with single HLA class I coated beads (A) or MOG_1–125_ coated beads (B) before being subjected to cell separation using an ARIA FACS-sorter (A) or magnet based purification (B). Frequency of B cells interacting with nominal antigens is shown before purification and in the positive and in the negative fraction. One representative out of three experiments with cells from different donors is shown.

We next tested whether CD19^+^ cells that interact with antigen-coated beads and those that can not be isolated. Using a FACS-sorter or antigen-coated on magnetic beads, we show that CD19^+^ cells can be efficiently depleted of CD19^+^ cells that interact with MOG_1–125_ -coated beads or HLA class I-coated beads (Figure 4). After depletion, the negative fraction did not contain any CD19^+^ cells able to interact with antigen-coated beads. In contrast, the positive fraction was enriched in CD19^+^ that interact with either MOG_1–125_ - or HLA class I-coated beads (Figure 4). We observed that FACS-sorter based strategy was efficient for enrichment when the BBR frequency was higher than 0.8% (initial frequency of HLA class I specific B cells 0.871% and 2.71%; post-sorting frequency 21.1% and 32.6% respectively; Figure 4A). However, we were not able to retrieve MOG_1–125_ specific B cells using FACS-sorter when the initial frequency was lower than 0.7% (initial frequency of MOG_1–125_ specific B cells 0.257%, 0.628% and 0.641%). For lower frequency, an alternative strategy based on the use of antigen coated on magnetic beads was more efficient (frequency of MOG_1–125_ BBR before purification: 0.295%, 0.335% and 0.69%; frequency of MOG_1–125_ BBR post-purification 23.5%, 25.4% and 32.5% respectively; Figure 4B).

### Immunized Kidney Allotransplant Recipients Exhibit an Increased Frequency of CD19^+^ B Cells Against HLA Class I Single Antigens Coated Beads

**Figure 5:**
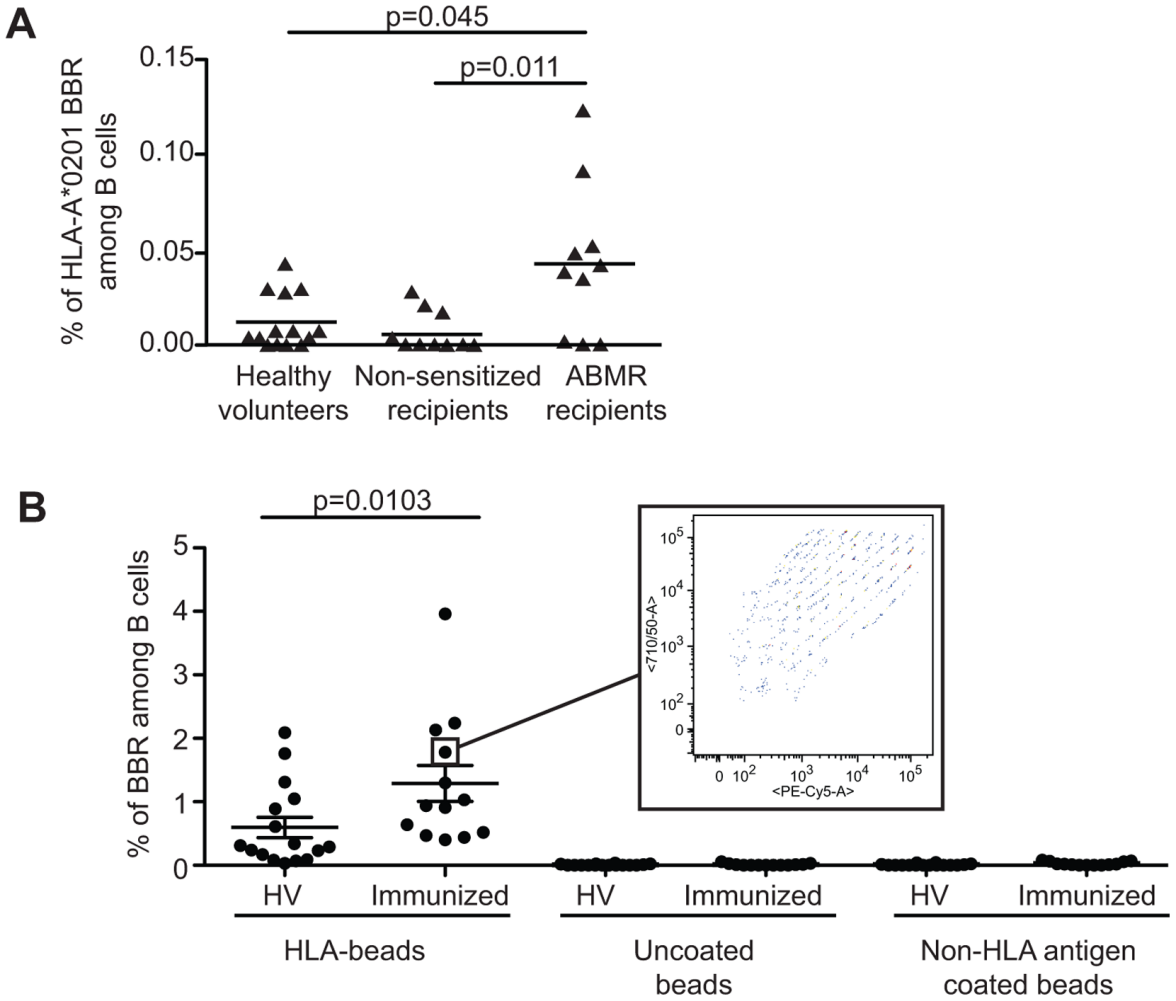
Enhanced frequency of anti-HLA B cell in immunized patients. **A.** Using single HLA-A*0201 coated beads, the frequency of B cells specific to HLA-A*0201 allele was assessed in the blood of sensitized transplant recipients with histologically proven antibody mediated rejection (ABMR; n = 10), non-sensitized stable transplant recipients (n = 9) and healthy volunteers (n = 14). Sensitized patients exhibit a significant increase in the frequency of HLA-A*0201 specific B cells compared to non-sensitized patients and healthy volunteers. p value are mentioned (Kruskall-Wallis follow by a Dunn’s post hoc test) **B.** B cells bound to single HLA class I coated beads (HLA-beads), to negative control (NC) and positive control (PC) were analyzed in HV (n = 16) and Immunized kidney recipients (n = 13). NC and PC beads were included by the manufacture in the single HLA class I kit. According to the manufacture, NC beads are beads saturated with ovalbumin and PC beads are coated with human IgG1. A broad range of single HLA class I were recognized as shown in the insert, a pattern observed for B cells from all tested patients. p value is indicated (Mann-Whitney test).

To show evidence of the potential usefulness of the method, we tested the B cell frequency in a context of over immunization against HLA antigens. Figure 5A shows that kidney recipients sensitized against HLA-A*0201 (n = 10; as shown by circulating anti-HLA-A*0201 antibodies interacting with the same beads) exhibited an increased frequency of HLA-A*0201-specific B cells compared to non-sensitized recipients (n = 8; p = 0.011) and to healthy volunteers (n = 14; p = 0.045).

Through varying the ratios of two fluorochromes within the bead core, it was then possible to assess up to 97 variables at the same time as detection of anti HLA allele products. Thus, we analyze the reactivity of blood B cells isolated from immunized recipients (n = 13) and from healthy volunteers (n = 16) against single HLA class I allele-coated beads. The single HLA class I allele coated beads were added in excess as exemplified by the detection of large amount of beads that had not interacted with B cells. The frequency of B cell recognizing beads coated with a single HLA class I allele was significantly higher in immunized recipients compared to healthy volunteers (mean±sem; 1.29±0.28 vs. 0.60±0.16 respectively, p<0.01; Figure 5B). Non-specific interaction was excluded as uncoated beads (NC) and non-HLA antigen coated beads were not recognized by healthy volunteers or immunized recipients. In addition, whereas the ability of B cells to recognize HLA class I antigen was restricted to a small fraction of B cells (<2% of total B cells), a very broad panel of HLA class I alleles were recognized (insert in Figure 5B), with a varying range of B cells interacting with each allele showing poly-specificity. Finally, the interaction between CD19 cells and HLA class I-coated beads was not influenced by the *in vitro* activation of the B cells. When purified CD19^+^ cells were activated *in vitro* for 4 days using an activation cocktail (anti-F(ab)’2, CD40L+Enhancer, ODN2006 and IL-2) the frequency of BBR remained unchanged (data not shown).

**Figure 6:**
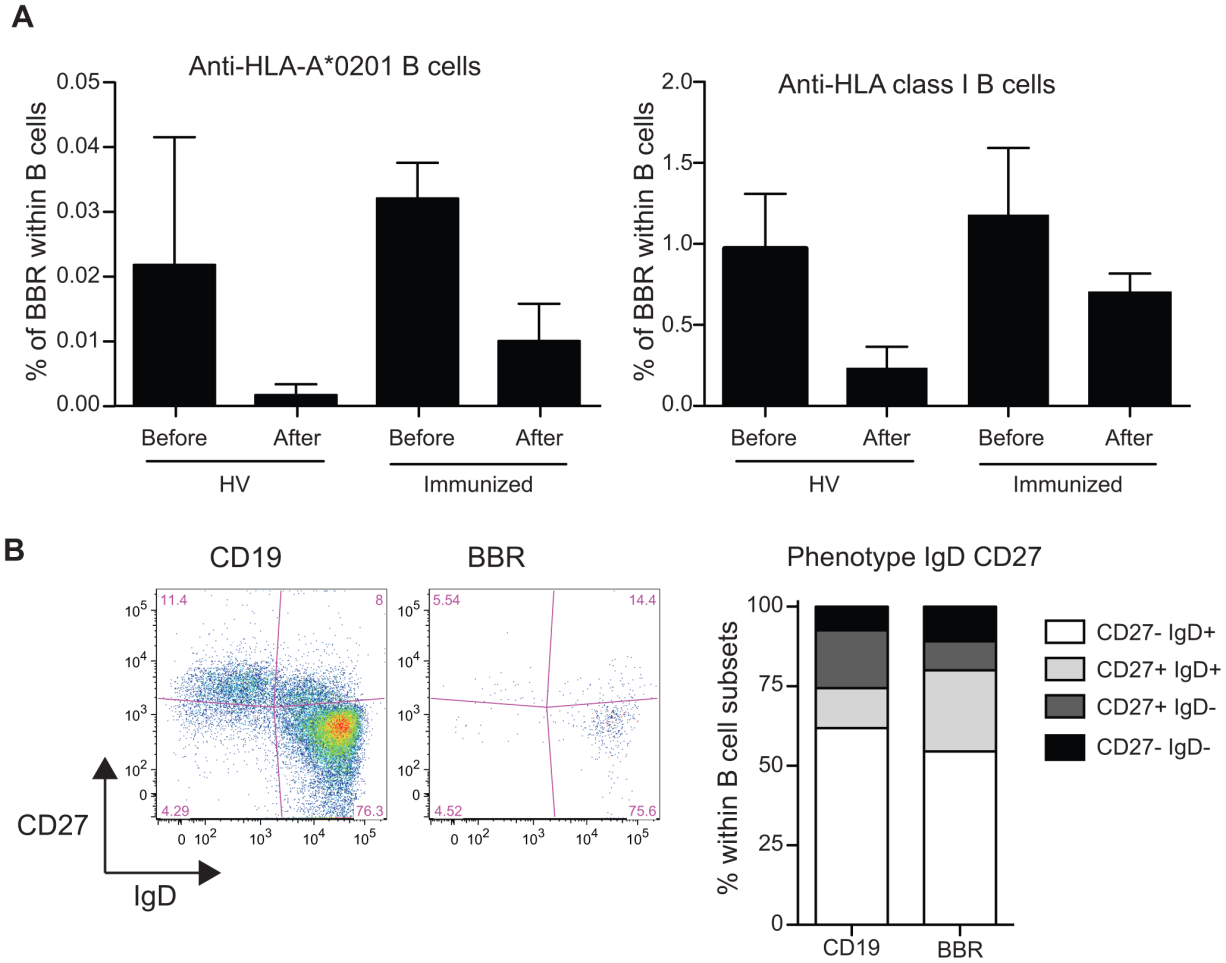
The interaction between CD19^+^ cells and HLA class I coated beads is not restricted by the HLA class I allele only and BBR are not restricted to the memory compartment. **A.** CD19^+^ cells were pre-incubated with a cocktail of HLA-A*0201 multimer (HLA-A*0201/MP_58–66_, HLA-A*0201/HCw1, HLA-A*0201/pp65) followed by the incubation with HLA Class I coated beads. Frequency of CD19^+^ cells specific of HLA-A*0201 coated beads and of HLA class I coated beads were analyzed before and after coincubation with the cocktail of HLA-A*0201 multimer for healthy volunteers (n = 6) and immunized patients (n = 3). Data are presented as mean ± sem. **B.** B cells were stained with anti-CD19, anti-CD27 and anti-IgD antibodies prior to incubation with single HLA coated beads. Phenotype of B cells and BBR were analyzed based on the expression of CD27 and IgD. 4 populations were identified (CD27^−^IgD^+^, naïve B cells; CD27^+^IgD^+^, non-switched memory B cells; CD27^+^IgD^−^, switched memory B cells; CD27^−^IgD^−^); CD27^−^IgD^−^ (late memory B cells). Representative phenotype of B cells and single HLA class I coated beads is shown as well as a summary of 5 immunized patients (mean).

Of note, whereas a high frequency of HLA-A*0201 CD19^+^ cells was observed when single antigen HLA-A*0201 coated beads were used, similar observation was not done when the panel of 97 different HLA class I coated beads was used in multiplex assay (data not shown). This apparent discrepancy is likely related to the presence within the test tube of a large number of different HLA class I molecules decreasing the number of specific events recorded for each antigen. To better approach the nature of allelic specificity, CD19^+^ cells purified from HV or immunized patients were pre-incubated with a cocktail of HLA-A*0201 multimers (HLA-A*0201/MP_58–66_, HLA-A*0201/HCw1, HLA-A*0201/pp65) before the incubation with HLA class I coated beads. Despite reactivity against HLA-A*0201 coated beads being strongly reduced after the pre-incubation with HLA-A*0201 multimers (Figure 6A), the frequency of B cells making rosettes against non-HLA-A*0201 molecules was similarly affected by the pre-incubation with HLA-A*0201 multimers (Figure 6A), confirming a significant level of polyreactivity in the circulating B cells when antigens with minor differences are tested.

### HLA Class I Specific CD19^+^ Cells from Patients Immunized Against HLA Antigens are not Restricted to the Memory Compartment

The phenotype of the B cells that recognized HLA class I antigens was analyzed by flow cytometry based on the co-expression of CD38 and IgD [26] or of CD24 and CD38 [27]. These classifications allow the identification of the successive cell development stages from naïve B cells to differentiated memory B cells. According to the analysis of co-expression of CD38 and IgD or CD24 and CD38, the phenotype of B cells interacting with HLA class I antigens and of those not was similar (data not shown). Next, we investigate in more details the co-expression of CD27 and IgD, markers that allow the discrimination of naïve mature (CD27^−^IgD^+^), class-switched memory (CD27^+^IgD^−^), non-switched memory (CD27^+^IgD^+^) and late memory B cells (CD27^−^IgD^−^) B cells. As for total B cells, the naïve mature phenotype (i-e CD27^−^IgD^+^) was the most frequent phenotype for B cells interacting with HLA class I antigens (mean±sem; 54.5±6.3 vs. 61.8±7.4; Figure 6B). B cells interacting with HLA class I antigens exhibited a higher proportion of non-switched CD27^+^IgD^+^memory B cells (mean±sem; 25.5±5.4 vs. 12.6±4.5 respectively; p = 0.0278; Figure 6B) and a lower proportion of class-switched memory (CD27^+^IgD^−^) B cells than the B cells that do not interact with HLA Class I antigen (mean±sem; 9.12±4.07 vs. 18.16±2.12 respectively; p = 0.0754; Figure 6B). The ratio of non-switched memory to class-switched memory B cells was ten times higher in HLA class I specific BBR compared to CD19 cells (mean±sem; 7.9±4.6 vs. 0.7±0.2 respectively; p = 0.03; Figure 6B).

## Discussion

In this paper, we describe a new strategy for measuring the frequency of antigen-reactive B cells. Taking advantage of beads that can be covalently linked to antigens and detected thanks to their fluorescent internal core, the reactivity of blood B cells against various antigens (alloantigens, self, viral and vaccine antigens) has been assessed in a transgenic mouse model, in healthy volunteers and in allo-immunized patients. Whereas the frequency of B cells against a pool of 97 HLA Class I molecules was similar to the levels obtained with albumin-coated beads, a substantial frequency of B cells interacting with self protein (MOG_1–125_) and high frequency for viral protein (EBNA1) or a vaccinated protein (TT) in healthy individuals could be observed. When this method was applied in patients with known immunizing histories (i.e; kidney allograft recipients with circulating antibodies against HLA antigens), a significant increase in the frequency of B cells specific to HLA class I molecules was observed. Of interest, the BBR phenotype was not biased toward a memory-switch phenotype, suggesting that different B cell populations contribute to this profile.

Great attention has been paid to the validation of this strategy. Using 8.18-C5 mice [15], we show that B cells specifically interact with MOG_1–125_ coated beads but not with beads coated with other antigens (pp65). Pre-incubation of B cells with soluble MOG_1–125_, but not with irrelevant CMV pp65 antigen, prevents the interaction between B cells and MOG_1–125_ -coated beads. Of note, a limited amount of beads can be used as an excess of beads was always evidenced by the beads that did not interact with B cells on the dot plot.

T and B-cell ELISPOT have also been used to measure committed B cell frequency against a given antigen. However, the ELISPOT assay does not measure the frequency of cells that actually interact with the antigen (which can for instance elicit an immune synapse for T cells or directly bind to antigen for B cells) but measures biological events such as cytokine release [28] or production of immunoglobulin after differentiation *in vitro* [29], [30], events that result from the interaction of the cells with the antigen. In this context, the estimated frequency is restricted to the cells that are able to be selectively stimulated by the antigen depending on read-out and thus may lead to an underestimation of the actual frequency of committed cells. In contrast, the BBR approach identifies all B cells actually interacting with a putative antigen through the BCR. Moreover, the BBR technique also allows the phenotyping of antigen specific B cells. Finally the flexibility of the system (the fluorescent beads can be coated with virtually any peptide or protein) and the unavailability of fluorescent molecules to the B cells (the fluorochromes are encapsulated within the internal core of the beads) are key advantages over the use of tetramer.

We studied the recipients of a mismatched kidney allograft with circulating anti-HLA Ab to prove that the method can identify an increase in circulating committed B cells following immunization. We show that kidney recipients who developed a humoral response against HLA antigens have also a significantly higher frequency of B cell forming rosettes with HLA-A*0201 (in a single HLA coated bead example) or HLA antigens (in a multiplexed bead example) than non-immunized recipients or normal individuals. Whether this expanded circulating alloreactive B cell pool plays a role in acute or chronic rejection of an allograft would be an important area for further study. These results suggest that what is observed in the periphery (blood) could reflect the presence of a similar population in the graft, conforming to the reported association of intra-graft B cells and rejection severity[31]–[33]. Studying the frequency of HLA committed B cells from the blood before and after transplantation and possibly from the graft would be of paramount importance in better understanding their role in rejection but also in tolerance mechanisms after transplantation. Furthermore, we show that usage of magnetic beads yields very efficient enrichment of specific BBR, which will allow more focused studies on their characteristics.

Within germinal centers, B cells undergo rounds of division and affinity maturation and high-affinity cells further differentiate into memory B cells or long-lived plasma cells. The preferential phenotype of BBR within unswitched B cells, and especially within naïve/mature B cells, suggests that the circulating antigen specific B cells do not undergo a germinal center formation but rather encompass the naïve B cell compartment. However, it is likely that the BBR population is a mixture of committed memory B cells and of naïve B cells with crossreactive/polyreactive BCR [24] as suggested, for instance, by the high frequency of blood B cells binding TT-coated beads – a situation where no antigen may remain and where ≈ 6% of B cells are committed against TT [34]. The monitoring of the levels of B cells with a BCR interacting with different viral proteins or with a protein used for vaccination may be also be worth considering.

Altogether, this study shows that precise identification of B cells committed against an individual antigen is possible in humans and that there is substantially high frequency of circulating B cells committed against more than one tested protein, confirming the low efficiency of the first checkpoint in B cell development. We suggest that this approach may allow further understanding of a number of pathological processes.
