# Correction: Characterization of Antigen-Specific B Cells Using Nominal Antigen-Coated Flow-Beads

**DOI:** 10.1371/journal.pone.0102845

**Published:** 2014-07-09

**Authors:** The PLOS ONE Staff

The second author’s name is listed incorrectly in the citation of the article. The correct name is: Annie Elong Ngono. The correct citation is: Degauque N, Elong Ngono A, Akl A, Lepetit M, Crochette R, et al. (2013) Characterization of Antigen-Specific B Cells Using Nominal Antigen-Coated Flow-Beads. PLoS ONE 8(12): e84273. doi:10.1371/journal.pone.0084273
